# Microbial Production of Bacterial Cellulose Using Chestnut Shell Hydrolysates by *Gluconacetobacter xylinus* ATCC 53524

**DOI:** 10.4014/jmb.2208.08022

**Published:** 2022-09-23

**Authors:** Jeongho Lee, Kang Hyun Lee, Seunghee Kim, Hyerim Son, Youngsang Chun, Chulhwan Park, Hah Young Yoo

**Affiliations:** 1 Department of Biotechnology, Sangmyung University, Seoul 03016, Republic of Korea; 2 Department of Bio-Convergence Engineering, Dongyang Mirae University, Seoul 08221, Republic of Korea; 3 Department of Chemical Engineering, Kwangwoon University, Seoul 01897, Republic of Korea

**Keywords:** *Gluconacetobacter xylinus*, bacterial cellulose, chestnut shell, food processing residue, enzymatic hydrolysates, acetic acid

## Abstract

Bacterial cellulose (BC) is gaining attention as a carbon-neutral alternative to plant cellulose, and as a means to prevent deforestation and achieve a carbon-neutral society. However, the high cost of fermentation media for BC production is a barrier to its industrialization. In this study, chestnut shell (CS) hydrolysates were used as a carbon source for the BC-producing bacteria strain, *Gluconacetobacter xylinus* ATCC 53524. To evaluate the suitability of the CS hydrolysates, major inhibitors in the hydrolysates were analyzed, and BC production was profiled during fermentation. CS hydrolysates (40 g glucose/l) contained 1.9 g/l acetic acid when applied directly to the main medium. As a result, the BC concentration at 96 h using the control group and CS hydrolysates was 12.5 g/l and 16.7 g/l, respectively (1.3-fold improved). In addition, the surface morphology of BC derived from CS hydrolysates revealed more densely packed nanofibrils than the control group. In the microbial BC production using CS, the hydrolysate had no inhibitory effect during fermentation, suggesting it is a suitable feedstock for a sustainable and eco-friendly biorefinery. To the best of our knowledge, this is the first study to valorize CS by utilizing it in BC production.

## Introduction

Bacterial cellulose (BC), a homopolymer of β-1,4-linked glucose, is a biodegradable natural polymer produced by bacteria, including *Gluconacetobacter*, *Azotobacter*, and *Pseudomonas* [1]. When compared with plant cellulose (PC), BC is gaining popularity for environmental and product quality reasons. Deforestation, an essential process in producing PC-based products, reduces the ecosystem’s ability to absorb existing carbon dioxide [2]. BC has outstanding properties, such as high-water absorption and retention capacity, high crystallinity and porosity, and strong biocompatibility [3, 4]. In particular, the high purity of BC eliminates the need for conventional PC processing steps that remove other plant components (*e.g.*, lignin) [5]. The global BC market was worth US$250 million in 2019 and is expected to rise to 570 million by the end of 2024 [6]. Despite expected growth trends in market size, the high cost of fermentation media for BC production (approximately 30% of total production cost) is identified as a limiting factor [7]. For the sustainable production of BC, it is necessary to use cost-effective media, such as biomass-derived media.

The biorefinery of food processing residues (FPRs), including shell biomass, is regarded as a sustainable strategy due to low cost and lack of competition with food materials [8, 9]. However, fermentation inhibitors derived from biomass compositions (*e.g.*, lignin and sugars) can be generated during the sugar conversion processes to produce fermentable sugars. Fermentation inhibitors are commonly produced by acid hydrolysis, but they can also be generated by alkali pretreatment and enzymatic hydrolysis of biomass [10]. Guragain *et al*. (2016) reported that increasing the concentration of the alkali pretreatment reagent (NaOH) increased the formation of acetic acid, formic acid, and phenolic compounds from sorghum [11]. Because the presence of inhibitors can impair microorganism fermentation performance, it was necessary to evaluate the suitability of biomass hydrolysates for use [10]. These evaluations were done in various studies by identifying fermentation inhibitors in hydrolysates and comparing fermentation performance with controls [12-15].

Previously, we prepared chestnut shell (CS) hydrolysates through alkali pretreatment and enzymatic saccharification [16]. In this study, CS hydrolysates were used as a medium for the BC-producing bacterial strain *Gluconacetobacter xylinus* ATCC 53524. Prior to using CS hydrolysates, potential inhibitors in the hydrolysates were identified to determine the presence of inhibitors that might contribute to the low BC production yield. Following that, to compare the fermentation performance of *G. xylinus* in CS hydrolysates with that in commercial glucose, BC concentrations were profiled during both fermentations. Finally, the surface morphology and chemical properties of the produced BC were analyzed.

## Materials and Methods

### Materials

Sodium hydroxide (NaOH), citric acid monohydrate, ammonium sulfate, dibasic potassium phosphate, magnesium sulfate heptahydrate, hydrochloric acid (HCl), and sulfuric acid (H_2_SO_4_) were purchased from Duksan Chemical (Korea). Acetic acid, formic acid, 5-hydroxymethylfurfural (5-HMF), furfural, Celluclast 1.5 L, Cellic CTec2, glucose, and corn steep liquor (CSL) were purchased from Sigma-Aldrich (USA). Yeast extract and peptone were purchased from Difco (USA). All chemicals and reagents used in this study were of analytical grade.

### Preparation of CS Hydrolysates

CS was purchased from Cheongmyeongyagcho (Korea). The CS hydrolysates were prepared according to our previous study [16]. First, the dried CS was pretreated under the following conditions: solvent, 1.9% (w/w) NaOH solution; solid loading, 100 g/l; temperature, 25°C; and reaction time, 2.8 h. Next, the pretreated CS was neutralized, dried, and then hydrolyzed with enzyme cocktail under the following conditions: Celluclast 1.5 L loading, 60 filter paper units/g biomass; Cellic CTec2 loading, 30 cellobiase units/g biomass; buffer, 50 mM sodium citrate buffer (pH 4.8); solid loading, 30 g/l; temperature, 50°C; agitation, 180 ×*g*; and reaction time, 72 h. Finally, the enzymatic hydrolysates from CS were concentrated to a target concentration of 40 g glucose/l hydrolysates. The prepared hydrolysates were analyzed for potential inhibitors before being used as carbon sources for the BC-producing strain.

### Pre-Culture Procedure for *G. xylinus*

The BC-producing strain used in this study was *G. xylinus* ATCC 53524. *G. xylinus* was pre-cultured for two days in YPD broth at 30°C and 150 ×*g*. The YPD medium contained (g/l): glucose 40, peptone 20, and yeast extract 10 at pH 6.0. To easily measure the optical density (OD) of cells, Celluclast 1.5 L (1 vol%) was added to the pre-culture medium, which did not produce bacterial cellulose [17]. Prior to addition, Celluclast 1.5 L was filtrated through a sterilized syringe filter with a pore size of 0.22 μm to prevent contamination. The pre-cultured suspension was centrifuged for 10 min at 13,000 ×*g*, and the pellets were resuspended in sterile deionized water to obtain a cell OD of 0.8 at 600 nm. This cell suspension was used in the main culture for BC production.

### BC Production and Purification

An 800 μl volume of the suspension was inoculated into 10 ml of the main medium and statically cultured under aerobic conditions in a 50 ml Erlenmeyer flask at 30°C for 96 h. The main medium was prepared according to the previously described protocol by Kim *et al*. (2020) with some modifications, and contained (g/l): glucose 40, CSL 20, ammonium sulfate 4, dibasic potassium phosphate 2, and magnesium sulfate heptahydrate 0.4 at pH 6.0 [15]. Among them, glucose was derived from CS (for the experimental group) or obtained from commercial glucose (for the control group). The produced BC was harvested at specific time points (48, 72, and 96 h) to profile the BC concentration (g/l). After harvesting, the wet BC pellicles were washed several times with distilled water (DW), heated in DW to 60°C for 2 h, and then soaked in 1 N NaOH at 30°C overnight to remove residual cells and medium [15]. The cleaned BC pellicles were neutralized with DW and then dried at 60°C overnight. All experiments were performed in triplicate and the results were presented as an average. The BC concentration was determined using Eq. (1):

$$\text{BC concentration (g/l) = }\frac{weight\;of\;dried\;BC\;(g)}{volume\;of\;culture\;medium\;(L)}$$ (1)

### Analytical Methods

Concentrations of glucose and potential inhibitors (acetic acid, formic acid, 5-HMF, and furfural) in CS hydrolysates were quantified using high-performance liquid chromatography (HPLC) analysis. The following conditions were set for HPLC analysis: column, Shodex SUGAR SH1011 H^+^ ion exclusion column (8 mm × 300 mm, Shodex, Japan); detector, refractive index detector (RID-10A, Shimadzu, Japan); mobile phase, 0.005 N H_2_SO_4_; flow-rate, 0.8 ml/min; column temperature, 50°C; and sample injection volume, 20 μl [18].

The surface morphology and chemical properties of dried BC (recovered at 96 h) were analyzed using scanning electron microscopy (SEM, Quanta FEG 250, FEI, USA) and Fourier-transform infrared (FTIR) spectroscopy (JASCO FTIR-4600), respectively.

## Results and Discussion

### Identification of Inhibitory Compounds in CS Hydrolysates

The presence of major fermentation inhibitors, such as acetic acid, formic acid, 5-HMF, and furfural, in CS hydrolysates was investigated to determine their use for BC production. Table 1 shows the composition of inhibitors in CS hydrolysates. Only 1.9 g/l of acetic acid was detected in CS hydrolysates concentrated at 40 g glucose/l. Acetic acid can be produced during the alkali pretreatment and enzymatic saccharification of biomass processes [19]. Acetic acid is produced specifically when the acetyl group of hemicellulose is released during this enzymatic saccharification process [20]. CS contains 2.4–5.9% (w/w) of hemicellulose, as previously reported [21]. Thus, residual acetyl groups in hemicellulose may have been released from the CS during enzymatic saccharification. For instance, Toquero & Bolado (2014) reported 3.6 g/l of acetic acid in wheat straw hydrolysates after NaOH (1%, w/w) pretreatment (100 g/l solid loading; 121°C temperature; and 1 h reaction time) and enzymatic hydrolysis [10].

If 1.9 g/l of acetic acid negatively affects *G. xylinus* fermentation, it must be removed from the hydrolysates prior to the fermentation process. However, we previously demonstrated that low concentrations of acetic acid (up to 2.0 g/l) improved the BC production yield [15]. Acetic acid can be utilized as a carbon source due to its close metabolic pathway to the tricarboxylic acid cycle in BC-producing bacteria strains [22, 23]. Clearly, numerous studies have demonstrated that acetic acid has a positive effect on BC production [24-27]. Based on these reports, we used the prepared CS hydrolysates as a fermentation medium for *G. xylinus* without any detoxification process.

### Microbial Production of BC Using CS Hydrolysates

**Fig. 1:**
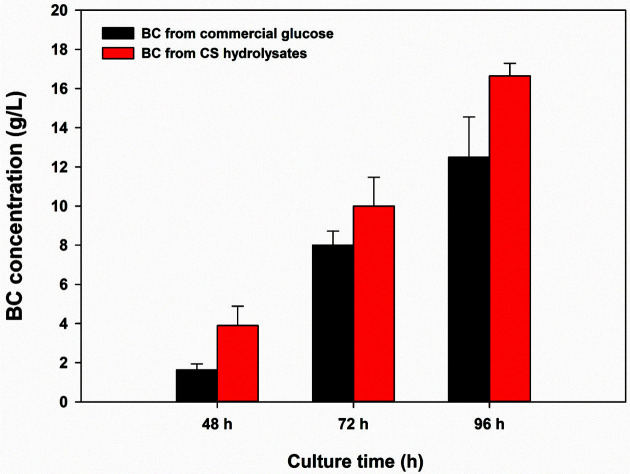
Profiles of BC concentration during fermentation by *Gluconacetobacter xylinus* ATCC 53524.

To evaluate the suitability of the CS hydrolysates for use, the fermentation performance of *G. xylinus* in the experimental medium (with CS-derived glucose) was compared with that in the control medium (with commercial glucose). Figure 1 shows the profiles of BC concentrations during 96 h of fermentation. In both groups, BC was biosynthesized as glucose was consumed (data not shown). According to Hur *et al*. (2020), *G. xylinus* synthesizes BC from glucose through several enzymes, such as glucokinase, phosphoglucomutase, UTP-glucose-1-phosphate uridylyltransferase, and bacterial cellulose synthase [28]. It is presumed that the microbial conversion of glucose (obtained from commercial glucose or derived from CS) into BC was possible due to the expression of these enzymes. The tendency to increase BC concentration was similar in both groups. After 48 h, the BC concentrations in the control and experimental groups were 1.6 g/l and 3.9 g/l (2.4-fold improved), respectively. After 96 h, the final concentrations of BC from commercial glucose and CS hydrolysates were 12.5 g/l and 16.7 g/l (1.3-fold improved), respectively. It is presumed that consumption of acetic acid as a carbon source may have a positive effect on BC production in *G. xylinus*. In fact, after 48 h, the acetic acid concentration in the experimental medium had dropped to 1.1 g/l (data not shown). Overall, the results of BC fermentation were better in the CS hydrolysate medium. Therefore, prepared CS hydrolysates are suitable raw materials that can be used directly as a BC production medium without the need for detoxification.

**Fig. 2:**
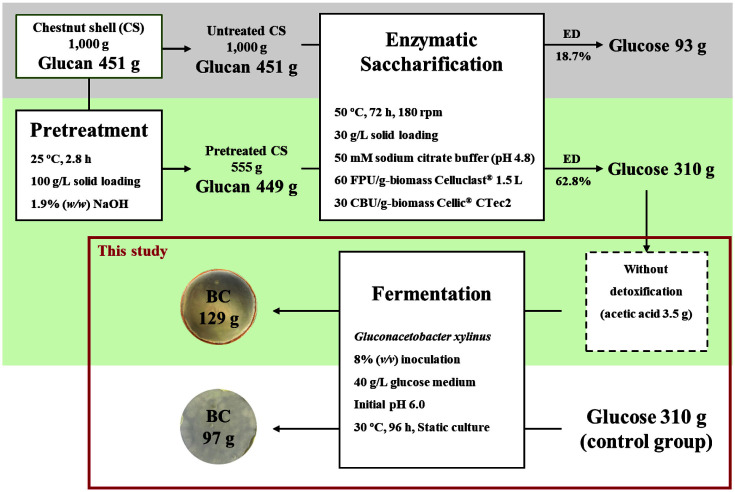
Mass balance for BC production from 1,000 g of CS.

The mass balance of BC production was calculated in the overall process using 1,000 g CS as a feedstock (Fig. 2). As described previously, we optimized an alkali pretreatment method to enhance glucose conversion from CS [16]. Alkali pretreatment is effective in removing lignin and consequently enhances the enzymatic conversion of cellulose to glucose [29]. As a result of alkali pretreatment, we achieved approximately 3.4-fold improved enzymatic digestibility under the determined conditions, and about 310 g glucose can be recovered from 1,000 g CS [16]. In recent literature on sugar conversion processes for CS, Eryasar-Orer and Karasu-Yalcin (2021) prepared CS hydrolysates through 1.25% sulfuric acid hydrolysis with a biomass loading of 10 g/100 ml, resulting in about 33.7 g/l xylose [30]. On the other hand, Morana *et al*. (2017) pretreated CS with an alkali reagent (5%aqueous ammonia), resulting in about 1.4-fold improved glucan content of CS, and then obtained CS hydrolysates through enzymatic hydrolysis [31]. The sugar conversion process can be designed differently depending on the biomass composition or target sugar product. Because of the high lignin content (about 41%) [31] and high glucan content (about 45%) [21] of CS, we performed alkali pretreatment. Finally, in this study, CS hydrolysate was successfully utilized for microbial BC production without detoxification, resulting in a 1.3-fold increase in BC production compared to that in the control group. The hydrolysates contain 310 g glucose and 3.5 g acetic acid, and based on 1,000 g CS, approximately 129 g BC can be produced.

In recent years, FPRs (*e.g.*, kiwifruit peel [32], citrus peel [33], pineapple waste [34], and wine pomace [35]) as well as lignocellulosic biomass (*e.g.*, *Miscanthus* [36], sweet sorghum [37], and pine tree [15]) have been actively studied and reported as a potential feedstock for BC production. Similarly, we attempted to valorize CS through microbial conversion to BC, and the results were promising. To realize CS as an industrial fermentation medium, scale-up of BC fermentation using CS hydrolysates should be performed and the application of BC should be studied. In this study, BC fermentation was performed on a lab-scale to evaluate the suitability of CS hydrolysates. Therefore, our future work will include continuous exploration of suitable FPRs as a feedstock for BC production, scale-up studies, and applied studies to valorize BC.

### Characterization of the Produced BC

**Fig. 3:**
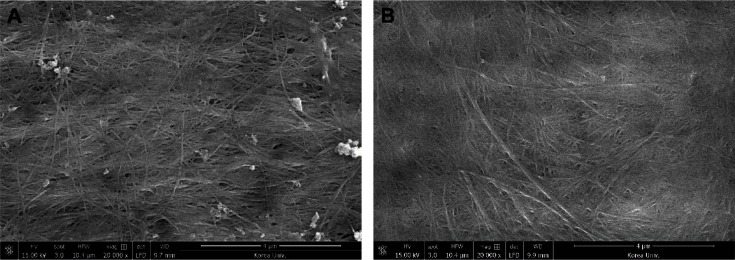
SEM images of BC produced from commercial glucose (**A**) and CS hydrolysates (**B**) after 96 h fermentation.

The surface morphology and chemical properties of dried BC derived from commercial glucose and CS hydrolysates were analyzed using SEM and FTIR. Fig. 3 shows SEM images of the BC samples, with those derived from glucose and CS hydrolysates shown to form ultrafine networks connected by numerous nanofibrils. Both BC samples were densely packed, and the images revealed a similar overall arrangement. However, the nanofibrils of CS hydrolysates-derived BC were observed to be relatively more densely arranged than those of the control group (Fig. 3B). These results visually confirm the improved BC production performance observed above in the experimental medium (Fig. 1).

**Fig. 4:**
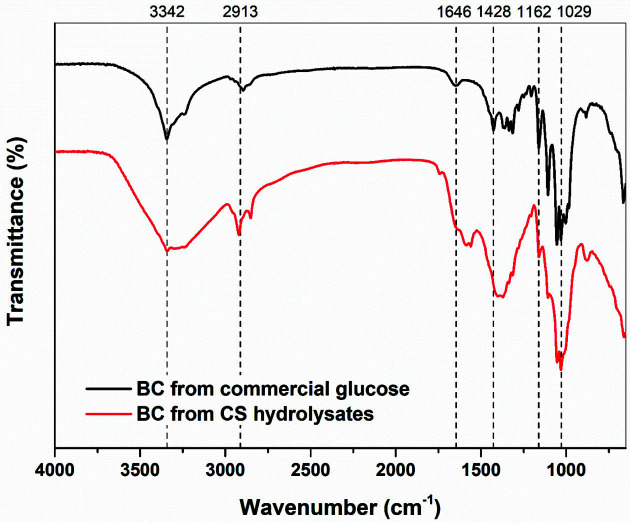
FTIR spectra of BC produced from commercial glucose and CS hydrolysates.

Fig. 4 shows the FTIR spectra of BC produced from commercial glucose and CS hydrolysates. Signature peaks in both were seen at 1029, 1162, 1428, 1646, 2913, and 3342 cm^-1^. Peaks at 1029 cm−1 are associated with cellulose C–O–C and C–O–H stretching vibrations [38]. The weak band at around 1162 cm−1 corresponded to the C–O–C asymmetric stretching [15]. The bands at 1428 cm^-1^ and 1646 cm^-1^ have been associated with symmetric and asymmetric COO stretching, respectively [39]. Peaks in the BC samples were found to be around 2913 cm^-1^ and 3342 cm^-1^, respectively, corresponding to C–H and O–H stretching vibrations [38]. Overall, the FTIR spectra revealed that the chemical properties of BC derived from commercial glucose and CS hydrolysates were similar. Based on the results for the surface morphology and chemical properties of BC samples, we emphasize that CS hydrolysates are a suitable and sustainable feedstock for BC production.

In this study, we successfully produced BC using CS hydrolysates through microbial conversion by *G. xylinus* ATCC 53524. Concentrated CS hydrolysates (40 g glucose/l hydrolysates) contained approximately 1.9 g/l acetic acid but had a positive effect on BC production. The concentration of BC from CS hydrolysates was 16.7 g/l, which was about 1.3-fold higher than that in the control group. The relatively densely arranged nanofibrils of the BC also confirmed this improvement. We therefore support the strategy of directly applying biomass hydrolysates containing an acceptable concentration of potential inhibitors to BC fermentation. In conclusion, this study provides a useful strategy for sustainable and eco-friendly BC production through microbial conversion of FPRs.

## Figures and Tables

**Table 1:** The composition of fermentation inhibitors in CS hydrolysates.

Compound	CS hydrolysates^a^
Acetic acid	1.9 g/l
Formic acid	ND
5-HMF	ND
Furfural	ND

CS: chestnut shell; 5-HMF: 5-hydroxymethylfurfural; ND: not detected.

^a^CS hydrolysates were concentrated to a target of 40 g glucose/l hydrolysates.
